# Endoscopic Resection of Pseudoarticulation as a Treatment for Bertolotti’s Syndrome

**DOI:** 10.7759/cureus.33397

**Published:** 2023-01-05

**Authors:** Eric Stein, Geoffrey D Panjeton, Sanjeev Kumar

**Affiliations:** 1 Anesthesiology and Critical Care, Saint Louis University School of Medicine, Saint Louis, USA; 2 Anesthesiology and Pain Medicine, University of Florida, Gainesville, USA

**Keywords:** endoscopic resection, pseudoarticulation, lumbosacral transitional vertebrae, interventional pain management, chronic low back pain, endoscopic spine surgery, bertolotti's syndrome

## Abstract

Bertolotti’s syndrome is described as lower back pain with the presence of lumbosacral transitional vertebrae (LSTV) associated with an articulation, pseudoarticulation, or full fusion of the transverse process to the sacrum and ilium. We present a unique case of the management of a 57-year-old woman with treatment-resistant lower back pain who underwent endoscopic resection of a pseudoarticulation related to LSTV. The patient underwent multiple treatment regimens without achieving satisfactory relief. These included physical therapy, sacroiliac (SI) joint injections, radiofrequency lesioning at multiple levels, and spinal cord stimulator placement. Relief with injection at the patient’s pseudoarticulation confirmed this as a contributor to the patient’s back pain. Interventional management of Bertolotti’s syndrome can include different modalities, most recently including minimally invasive surgical techniques. This patient experienced partial relief of lower back pain after undergoing minimally invasive resection of the pseudoarticulation. This case demonstrates the benefit of a minimally invasive resection of this anatomic abnormality in a patient who has undergone previous treatments. Isolating this anomaly as a source of pain is necessary to ensure a favorable response and prevent needless surgery.

## Introduction

Bertolotti’s syndrome refers to the presence of a lumbosacral transitional vertebra (LSTV) that causes lower back pain. The transverse processes of the fifth lumbar vertebrae (L5) have articulation or fusion with the first sacral vertebrae (S1) [1]. Occasionally, the transverse process may also fuse with the sacral ala. There is a wide range of estimated prevalences, but the mean reported prevalence is 12% [1]. The algorithmic approach to the management of Bertolotti’s syndrome is similar to that of lower back pain, beginning with conservative measures, progressing to percutaneous interventions, and finally surgical procedures [2,3]. We present the case of a 57-year-old woman with Bertolotti’s syndrome who underwent endoscopic resection of left L5-S1 pseudoarticulation.

## Case presentation

The patient was a 57-year-old woman with an extensive history of progressively worsening chronic low back pain that had been largely treatment-resistant. Her pain had been present for several years, and she did not note any inciting events or current triggers. The pain involved the lumbosacral region, with a referral down her left leg. She did not present with any other neurologic sequelae. She describes the pain as a constant 8/10 that was throbbing, cramping, and shooting in nature. All movements elicited pain. The FABER (Patrick’s) test was positive. The pain was exacerbated by prolonged sitting and any physical activity. It caused an impairment in her daily functioning. She had undergone many different conservative treatment modalities, including a multimodal pharmacologic regimen consisting of meloxicam and duloxetine, and had tried muscle relaxants in the past with minimal effect. Physical therapy had also been attempted on two separate occasions for six and eight weeks with minimal durable relief. Additionally, she had been performing home isotonic and isometric stretching exercises with marginal success. Interventional modalities included steroid injections, lumbar medial branch nerve radiofrequency ablations, and spinal cord stimulation, as outlined in Table 1. Regarding the placement of a spinal cord stimulator, this was done due to concerns about chronic radiculopathy early in the workup of this patient. These interventions resulted in varying degrees of success.

**Table 1:** Previous interventions

Procedural treatments (listed in descending chronological order)	Patient response
Bilateral sacroiliac joint (SIJ) injections: 2018	50% relief for two months
Nevro spinal cord stimulator placement: two percutaneous leads (2019)	Not reported
Left sacroiliac joint (SIJ) steroid injection: 2020	No relief
Bilateral L3-4 transforaminal epidural steroid injection (TFESI): 2020	Not reported
Bilateral L3-5 medial branch nerve radiofrequency ablation: 2020	80% relief for seven months
Bilateral L3-4 transforaminal epidural steroid injection (TFESI): 2021	Significant relief for one week
Bilateral sacroiliac joint (SIJ) injections: 2021	No relief
Bilateral greater trochanteric bursa injections: 2021	70% relief for three weeks
Bilateral L3-5 medial branch nerve radiofrequency ablation: 2021 (Figure 1A)	40% relief for two days
Bilateral L5-S1 pseudoarticulation steroid injection: 2021	Right side: 90% relief for two days. Left side: 80% relief for two days

**Figure 1:**
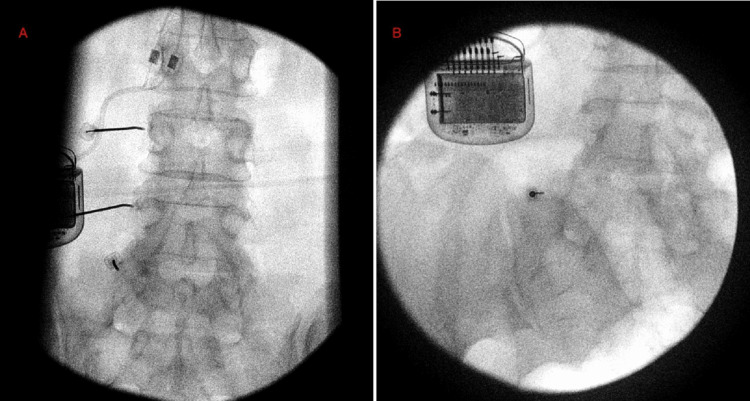
(A) Left L3-4 medial branch nerve radiofrequency ablation procedural image; (B) Operative image at left L5-S1 pseudoarticulation

**Figure 2:**
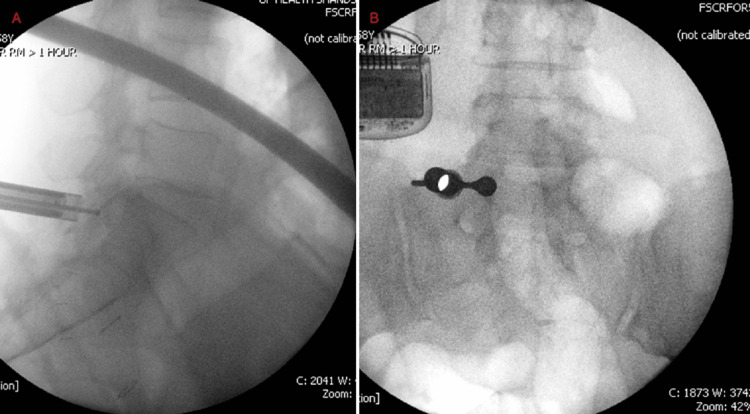
(A) Lateral image confirming complete resection of pseudoarticulation; (B) AP image of pseudoarticulation resection

Despite these therapies, our patient continued to have chronic low back pain. Given her responses to steroid injections at the pseudoarticulations, it was confirmed that these were significant contributors to her pain. After a discussion with the patient, she decided to undergo endoscopic resection of the left pseudoarticulation. The patient underwent general anesthesia and was placed in the prone position. General anesthesia was utilized to maximize patient safety and comfort during the procedure. Neuromonitoring was utilized during the procedure to minimize the risk of nerve damage. Intraoperatively, the left pseudoarticulation was identified under fluoroscopy. After anesthetizing the entrance site lateral to the pseudoarticulation (Figure 1B), a horizontal incision was made using a #11 blade, and blunt dissection was performed through to the thoracodorsal fascia. A dilator and an endoscope were placed. Soft tissue was removed using bipolar cautery and a pituitary rongeur until the osseous target was visible. The pseudoarticulation was shaved down and drilled through with a Kerrison rongeur and diamond bit drill. Complete decompression of the pseudoarticulation was confirmed via lateral and AP fluoroscopic views (Figure 2). Hemostasis was achieved, and the patient was taken to the recovery area without any complications. In the recovery area, she reported a decrease in her pain score from 7/10 to 4/10. At her initial follow-up appointment, the patient noted a mild improvement in her daily function and a decrease in her pain. She has noted sustained improvement in her pain at subsequent follow-up appointments and has not required any further interventions.

## Discussion

Bertolotti first described the association between congenital lumbosacral transitional vertebrae (LSTV) and lower back pain in 1917 [4]. LSTVs are associations or fusions of the fifth lumbar vertebrae with the sacrum (S1). The exact cause of lower back pain with Bertolotti’s syndrome is not fully understood. However, there is agreement that the presence of LSTV results in restricted movement between the L5 and S1 vertebrae [3]. As a result, the immediate cranial segment is more mobile and has degenerative changes. The decreased mobility in the articulation segment is thought to increase mobility and thus instability in the segment above. As Louma et al. found, the presence of LSTVs is associated with a statistically significant risk of degenerative changes in segments above the LSTV; these changes include increased rates of disc protrusion, disc degeneration, facet degeneration, and nerve root and canal stenosis [5,6]. However, they did not find a relationship between the presence of LSTVs and lower back pain. In a prospective cohort study by Taskaynathan et al., LSTVs were associated with statistically significant increases in clinical signs and lower back pain [7]. Additional findings include decreased sacral height and decreased caliber of the iliolumbar ligament in these patients [1].

The Castellvi scoring system is used to classify Bertolotti's syndrome subtypes. This system accounts for the degree of articulation between the segments as well as the laterality of the LSTV(s). This system uses imaging to classify LSTVs based on their osseous features and bilaterality. The addition of a or b indicates unilaterality or bilaterality, respectively. Type I describes an enlargement of the L5 transverse process that is greater than 19 mm. Type II describes an enlarged transverse process that has an incomplete articulation with the sacrum. Type III describes an enlarged transverse process with a complete articulation with the sacrum. Lastly, type IV describes the presence of both a type IIa and a type IIIa transverse process [8]. The vast majority of LSTVs involve a pseudoarticulation rather than a complete sacralization [9].

There is no specific consensus regarding the algorithmic management of patients with LSTVs and lower back pain, which constitute Bertolotti’s syndrome. Like other causes of low back pain, Bertolotti’s syndrome is managed first with conservative treatments. To localize the lower back pain to the LSTV, anesthetic blocks with or without steroids are utilized. If a patient has relief, further invasive management is indicated following the treatment algorithms proposed by Almeida et al. and Li et al. [2,3]. Radiofrequency ablation is typically the next step and can provide a less invasive option. Surgical options are also available, specifically fusion or resection of the LSTV. Santavaria et al. compared these two modalities by studying 16 patients with clinical and radiographic evidence of Bertolotti’s syndrome. Of these patients, eight underwent posterolateral fusion, and eight underwent surgical resection. After nine years, 10 of the 16 patients had improvement in pain with no difference between the two modalities [10]. Of note, these patients performed slightly better than a group treated conservatively. Recently, minimally invasive methods have been used as an alternative to traditional spine surgeries [11,12]. In a case series by Li et al., seven patients with Bertolotti’s syndrome and their responses to minimally invasive resection were followed. Three of the seven patients studied had complete pain relief, two had reduced pain, and two had initial relief followed by pain recurrence [3]. Additionally, three of six patients with radicular pain experienced resolution of this symptom.

**Figure 3:**
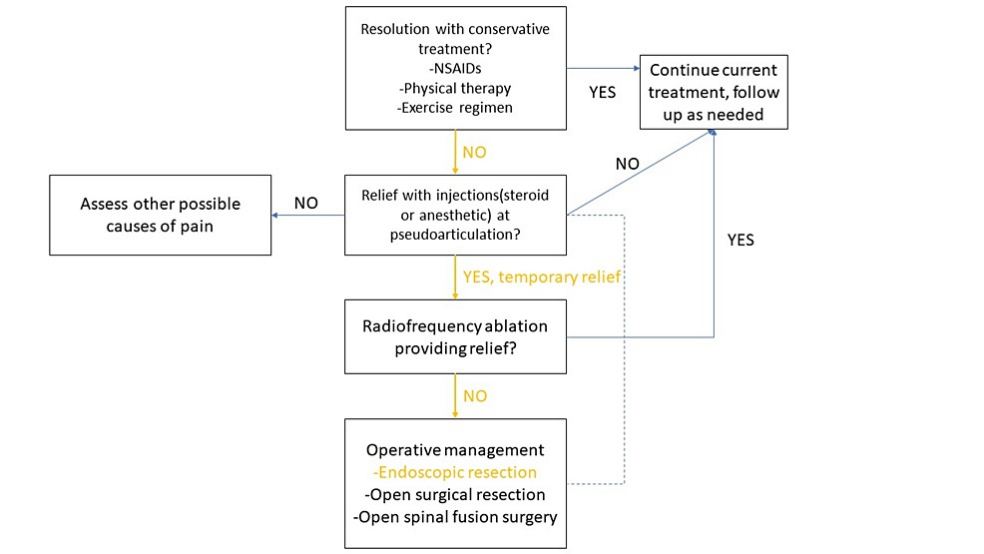
Treatment algorithm The algorithm was modified from Almeida et al. and Li et al. [2,3].  Pathway progression through the algorithm is shown in yellow.

**Figure 4:**
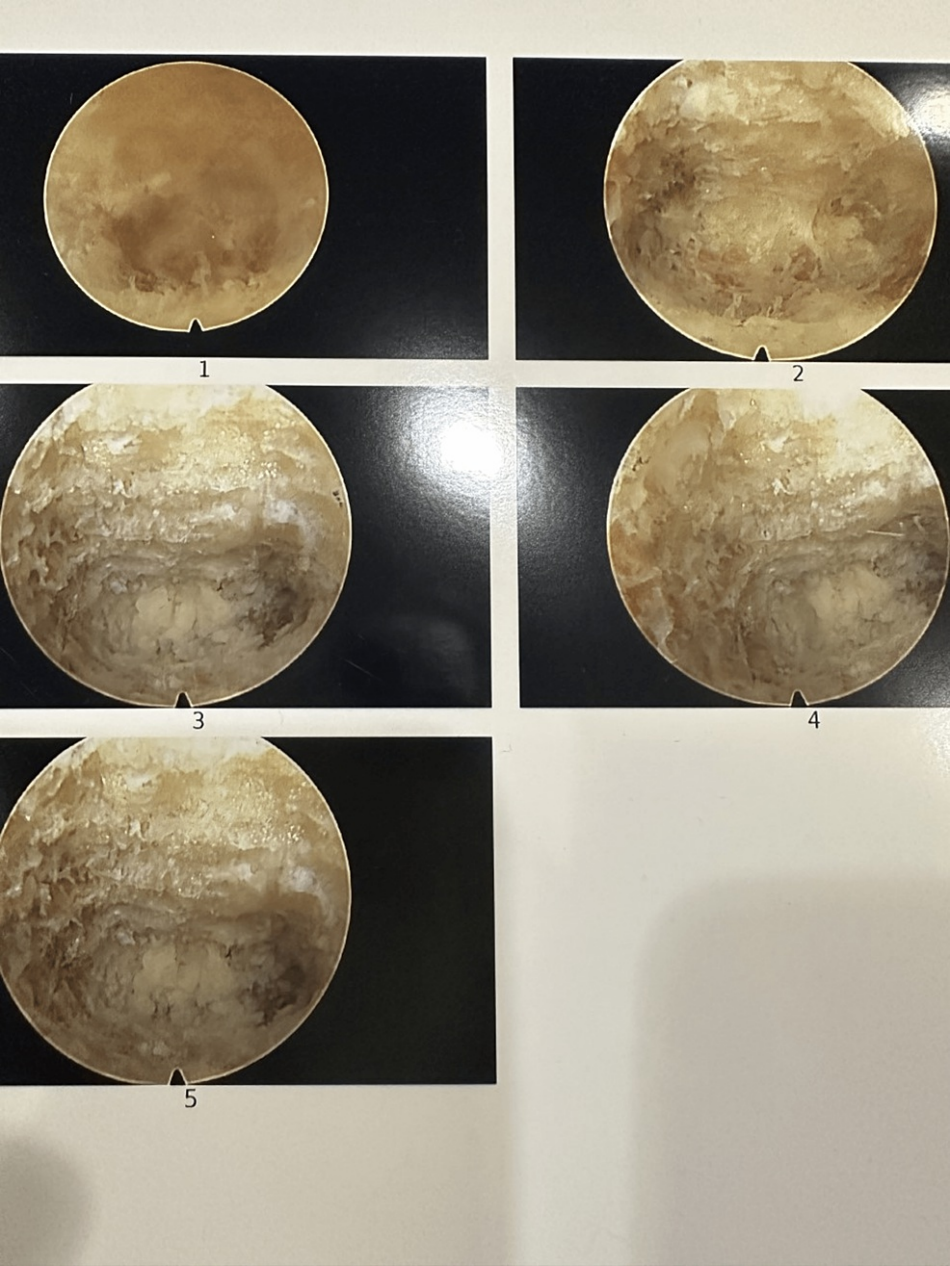
LSTV resection Image 1 shows LSTV as viewed through an endoscope. Image 2 shows a partial resection. Images 3-5 show complete osseous target resection with visible underlying retroperitoneal fat.

This patient was diagnosed with Castellvi IIb Bertolotti’s syndrome and experienced minimal relief with conservative modalities. Her progression through a treatment algorithm is detailed in Figure 3. The benefit from prior injections at the bilateral pseudoarticulations represented their role as pain generators in this patient. After a failed radiofrequency ablation, she opted for a minimally invasive resection of the left LSTV. We present one of the few cases of bilateral LSTV-associated pain treated with a minimally invasive approach. Since performing this first case of LSTV, we have continued to improve our technique. We have now resected along the entire length of the pseudoarticulation. Figure 4 shows endoscopic images of resection with this refined technique. As noted, our patient experienced partial, but not complete, pain relief. Subsequent patients treated with the refined technique outlined above have experienced complete pain relief. This case demonstrates that minimally invasive management can be an option for patients who have exhausted other treatment modalities. Numerous interventions targeting a variety of etiologies reflect the challenge of diagnosing this often-overlooked pathology. Further study of outcomes after a minimally invasive resection is needed.

## Conclusions

This case highlights a minimally invasive approach to address lower back pain due to LSTVs. We also highlight our patient's progress through a treatment algorithm for Bertolotti’s syndrome. Endoscopic resection of an LSTV can be performed for patients with treatment-resistant Bertolotti’s syndrome. This treatment provided partial relief and improved function for the patient. Isolation of the anomaly as a source of pain is necessary to prevent unnecessary interventions. In our experience, extensive resection along the entire length of the pseudoarticulation provides more complete pain relief.
